# Requirement of Osteopontin in the migration and protection against Taxol-induced apoptosis via the ATX-LPA axis in SGC7901 cells

**DOI:** 10.1186/1471-2121-12-11

**Published:** 2011-03-16

**Authors:** Rihua Zhang, Jing Wang, Shijie Ma, Zuhu Huang, Guoxin Zhang

**Affiliations:** 1 Department of Gastroenterology, the First Affiliated Hospital of Nanjing Medical University, Nanjing 210029, China; 2 Department of Infectious Disease, the First Affiliated Hospital of Nanjing Medical University, Nanjing 210029, China

## Abstract

**Background:**

Autotaxin (ATX) possesses lysophospholipase D (lyso PLD) activity, which converts lysophosphatidylcholine (LPC) into lysophosphatidic acid (LPA). The ATX-LPA signaling axis has been implicated in angiogenesis, chronic inflammation and tumor progression. Osteopontin (OPN) is an important chemokine involved in the survival, proliferation, migration, invasion and metastasis of gastric cancer cells. The focus of the present study was to investigate the relationship between the ATX-LPA axis and OPN.

**Results:**

In comparison with non-treated cells, we found that the ATX-LPA axis up-regulated OPN expression by 1.92-fold in protein levels and 1.3-fold in mRNA levels. The ATX-LPA axis activates LPA2, Akt, ERK and ELK-1 and also protects SGC7901 cells from apoptosis induced by Taxol treatment.

**Conclusions:**

This study provides the first evidence that expression of OPN induced by ATX-LPA axis is mediated by the activation of Akt and MAPK/ERK pathways through the LPA2 receptor. In addition, OPN is required for the protective effects of ATX-LPA against Taxol-induced apoptosis and ATX-LPA-induced migration of SGC7901 cells.

## Background

Autotaxin (ATX), also known as phosphodiesterase-I alpha (PD-I alpha) or ecto-nucleotide pyrophosphatase/phosphodiesterase 2 (NPP2 or ENPP2), is overexpressed in various tumors, including gastric cancer, ovarian cancer [1], breast cancer [2]. ATX has lysophospholipase D (lysoPLD) activity, which converts lysophosphatidylcholine (LPC) into lysophosphatidic acid (LPA) [3]. LPA levels have been reported to be elevated in diverse physiological and pathological conditions including pregnancy, high cholesterol diet, and ovarian cancer [4]. The biological outcome of ATX activity depends on the local availability of its substrate LPC [5]. The ATX-LPA signaling axis has been implicated in angiogenesis, chronic inflammation, fibrotic diseases and tumor progression, making this system an attractive target for therapy [6]. LPA elicits a variety of cellular biological responses through LPA receptors, which are a family of seven-transmembrane G protein-coupled receptors (GPCRs), including LPA1, LPA2, LPA3, and LPA4 [7]. LPA1-LPA3 receptors are expressed in various combinations in almost every tissue throughout the body, while LPA4 receptor is not widely expressed in human tissues [8]. LPA enhances the migration of gastric cancer cells by mediating the location of RhoA [9].

Osteopontin (OPN), a glycophosphoprotein cytokine, plays an important role in both physiological and pathological processes, such as cell adhesion, chemotaxis, protection of apoptosis, invasion and migration [10]. Recent studies have demonstrated that OPN is highly expressed in gastric cancer tissues compared to their surrounding gastric mucosa tissues [11]. In this study, we investigated the relationship between ATX-LPA axis and OPN in the human gastric cancer cell line, SGC7901 cells.

## Results

### LPA and ATX/LPC induced OPN expression in SGC7901 cells

**Figure 1:**
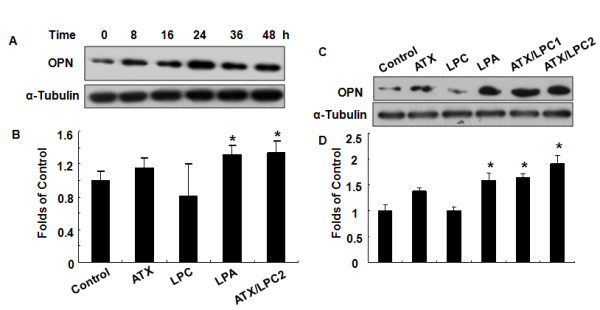
**LPA and ATX/LPC induce OPN expression in SGC7901 cells**. (A) Time course of OPN stimulation utilizing ATX (50 ng/ml) + LPC (20 μM) (ATX/LPC2). OPN expression was examined in SGC7901 cells by Western blot and real time-PCR. SGC7901 cells were treated with ATX, LPC, LPA, ATX/LPC1, ATX/LPC2 or DMEM/0.1% BSA. Total protein and RNA was extracted for Western blot (C) and real time -PCR (B), respectively. Figure1 D showed the densitometric ratio of OPN protein/α-tubulin. Statistical analysis was performed using Student's t test. *p < 0.05. The ATX/LPC concentration curve [4] for stimulated motility provides a justification for the concentrations of ATX/LPC chosen throughout the manuscript. LPA or ATX/LPC stimulates OPN production in additional cell lines (eg, SMMC7721 in our study: ATX-LPA axis induces expression of OPN in hepatic cancer cell SMMC7721) as well as in SGC7901 cells.

We first investigated the relationship between OPN and the ATX-LPA axis by examining the expression of OPN in SGC7901 cells treated with or without ATX, LPC, LPA, and ATX/LPC. Figure 1A shows a time course of OPN stimulation utilizing ATX (50 ng/ml) + LPC (20 μM) (ATX/LPC2) and a better time point of 24 hours. We found that LPA, ATX/LPC1 and ATX/LPC2 induced an increase in OPN expression in both protein (Figure 1C) and mRNA (Figure 1B) levels. However, ATX or LPC alone did not induce significant changes in OPN expression, suggesting that the increased OPN expression was induced by LPA, which was converted by ATX and LPC. Compared to non-treated cells, OPN expression induced by LPA, ATX/LPC1 and ATX/LPC2 was 1.58-fold, 1.63-fold and 1.92-fold, respectively in protein levels (Figure 1C); and 1.3-fold in mRNA levels (Figure 1B) (P < 0.05).

### ATX/LPC-regulated OPN expression was mediated by LPA2 receptor and activation of Akt and MAPK/ERK

**Figure 2:**
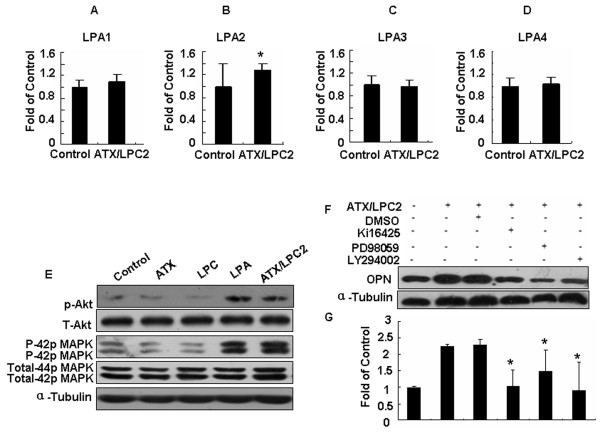
**ATX/LPC-regulated OPN expression is mediated by LPA2 receptor and activation of Akt and MAPK/ERK**. SGC7901 cells were exposed to ATX, LPC, LPA, ATX/LPC2 or DMEM/0.1% BSA for 24 hours or 30 min, and then total RNA and protein were extracted for real time-PCR (A-D) and Western blot (E, F), respectively. (E) Immunoblotting was performed to detect the phosphorylation status of ERK and Akt. Total ERK and Akt levels were assessed as control. (F) The effects of the LPA inhibitor, ERK inhibitor and Akt inhibitor on ATX/LPC2-induced OPN expression in SGC7901 cells. SGC7901 cells were treated with Ki16425, PD98059, LY294002 or DMSO (0.1%; a solvent control) for 45 min prior to ATX/LPC2 treatment. 24 hours following stimulations, OPN expression was measured by Western blot analysis. All experiments were performed in triplicate and a representative result is shown here. Figure 2G showed the densitometric ratio of OPN protein/α-tubulin. Statistical analysis was performed using Student's t test. *p < 0.05.

To determine whether the LPA receptor is responsible for LPA signal transduction, SGC7901 cells were treated with ATX, LPC, LPA, ATX/LPC2 for 18 hours, and the expression of LPA receptors was detected by real time-PCR. Our results suggest that the expression of LPA1, LPA2, LPA3 and LPA4 receptors were all found in SGC7901 cells. However, the expression of the LPA2 receptor was increased upon treatment with ATX/LPC2, suggesting that the LPA2 receptor might be the predominant receptor mediating LPA-induced OPN expression in SGC7901 cells (Figure 2A-D).

To further explore the mechanisms underlying LPA-induced OPN expression, we focused on the mitogen activated protein kinase (MAPK) and Akt signaling pathways. SGC7901 cells were treated with the reagents described above for 30 min, and total protein was extracted by Western blot to detect phosphorylation of either ERK or Akt. Total ERK and Akt levels were assessed as controls. We found that LPA and ATX/LPC2 induced both ERK and Akt phosphorylation in SGC7901 cells (Figure 2E). We treated SGC7901 cells with ATX/LPC2 in the presence of various inhibitors including the LPA receptor inhibitor, Ki16425; ERK inhibitor, PD98059; and PI3kinase inhibitor, LY294002 to examine the involvement of the LPA receptor, Akt and ERK in LPA or ATX/LPC2-induced OPN expression. Our results indicated that ATX/LPC-induced OPN expression in SGC7901 cells was largely reduced by the previously mentioned inhibitors (Figure 2F and 2G), suggesting that ATX/LPC-induced OPN expression is mediated by the LPA2 receptor as well as the activation of ERK and Akt.

### Determination of Elk-1 activities in SGC7901 cells

**Figure 3:**
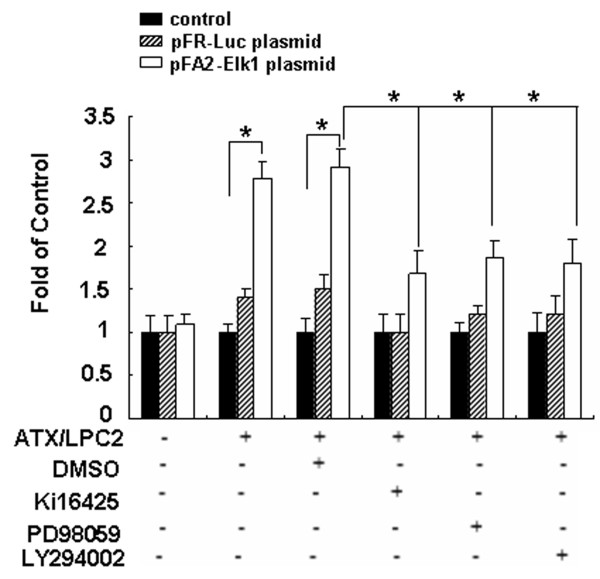
**Determination of Elk-1 activities in SGC7901 cells**. SGC7901 cells were transfected with either the pFR-Luc plasmid (reporter plasmid), or the pFA2-Elk1 plasmid (fusion trans-activator plasmid). Cells were then treated with ATX/LPC2, in the absence or presence of DMSO, Ki16425, PD98059, LY294002, harvested and the luciferase activity was measured. Statistical analysis was performed using Student's t test. *p < 0.05.

Transfection of SGC7901 cells with either the pFR-Luc plasmid (reporter plasmid), or the pFA2-Elk1 plasmid (fusion trans-activator plasmid) allowed us to gain a better understanding of the ATX-OPN signaling pathways. Cells were then treated with ATX/LPC2, DMSO, Ki16425, PD98059, and LY294002. Luciferase activity of pFA2-Elk1stimulated by ATX/LPC2 was approximately 2.7 folds higher compared with that of the non-treated control cells. Nevertheless, in the presence of Ki16425, PD98059, or LY294002, the pFA2-Elk1 activities induced by ATX/LPC2 were reduced by 1.67-fold, 1.87-fold and 1.79-fold, respectively (Figure 3). These reductions showed convincing evidence that the LPA receptor, ERK, and Akt were partially required in these processes.

### Requirement of OPN in migration induced by the ATX-LPA axis protected against Taxol-induced apoptosis in SGC7901 cells

**Figure 4:**
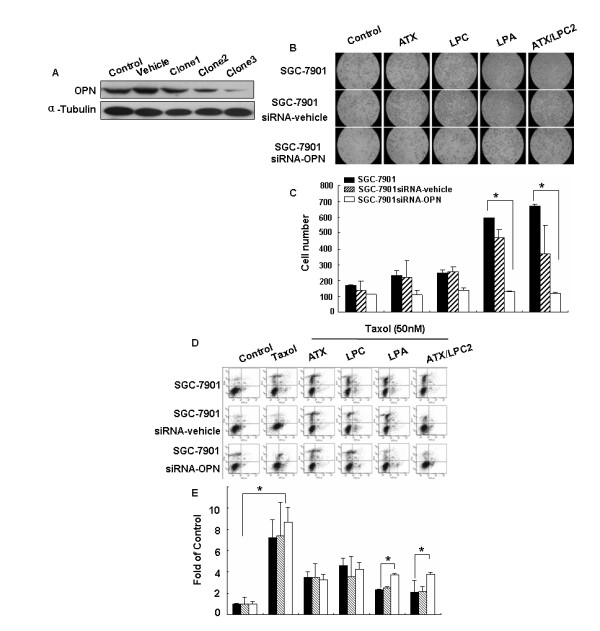
**Requirement of OPN in migration induced by ATX-LPA axis protects against Taxol-induced apoptosis in SGC7901 cells**. (A) Verification of siRNA knockdown of the OPN protein by Western blot showed a significant reduction of OPN protein in clone3 (SGC7901-siRNA-OPN). (B) The effect of OPN on ATX-LPA-induced migration was assayed using a Transwell assay. Either SGC7901, SGC7901-siRNA-vehicle, or SGC7901-siRNA-OPN cells (1 × 10^5^) were added in the upper chambers of the transwells, incubated in DMEM/0.1% BSA or medium containing ATX, LPC, LPA, ATX/LPC2 and allowed to migrate for 48 hours at 37°C. Migrated cells on the lower chamber were quantified. (D) Flow cytometric analysis of apoptosis: SGC7901, SGC7901-siRNA-vehicle, or SGC7901-siRNA-OPN cells were treated with Taxol (50 nM) in the absence or presence of each group: ATX, LPC, LPA, ATX/LPC2 or DMEM/0.1% BSA for 24 hours. Apoptosis was analyzed by flow cytometry. Data shown in Figure 4C and E represents the mean ± SD from three individual experiments. Data are mean ± SD of triplicate determinations. Statistical analysis was performed using Student's t test. *p < 0.05.

We established OPN-deficient SGC7901 cell lines through the introduction of OPN siRNA (clone1, clone2, clone3) to better understand the role of OPN in the ATX-LPA axis. SGC7901-vehicle was used as control. OPN expression was significantly down-regulated in clone3, which was then used in our study (Figure 4A).

A transwell-migration assay was performed to further investigate the biological functions of OPN knockdown in SGC7901 cells, and to detect LPA-induced migration in SGC7901-siRNA-OPN cells. LPA and ATX/LPC2 significantly promoted migrations of SGC7901 cells from either 169 cells to 596 cells (LPA) or to 670 cells (ATX/LPC2); from either 135 cells to 473 cells (LPA) or to 369 cells (ATX/LPC2) in SGC7901-siRNA-neg cells; however, no significant effect was found on the migrations of SGC7901-siRNA-OPN cells, from either 111 cells to 130 cells (LPA) or to 116 cells (ATX/LPC2) (Figure 4B, C), suggesting that OPN is required in the migration of SGC7901 cells induced by LPA or ATX/LPC.

ATX has been reported to protect Taxol-induced apoptosis in MCF-7 breast cancer cells and MDA-MB-435 melanoma cells [12]. We investigated whether the ATX-LPA axis also protected against Taxol-induced apoptosis in SGC7901 cells. Using flow cytometry, we found that treatment of SGC7901 cells with 50 nM Taxol resulted in a 64% increase in apoptosis. In comparing with the controls, either LPA alone, or ATX/LPC2 inhibited Taxol-induced apoptosis to 23.4% (LPA) or 21% (ATX/LPC2) in SGC7901 cells. Interestingly, in SGC7901-siRNA-OPN cells (clone3), LPA or ATX/LPC2 inhibited Taxol-induced apoptosis to 37% (LPA) or 37.7% (ATX/LPC2) (Figure 4D and 4E). The protective effect of LPA decreased to 58.1%. These results suggested that OPN is required in each of the processes and either LPA or ATX/LPC had a significant protective effect against Taxol-induced apoptosis.

## Discussion

Gastric cancer is the fourth most common cancer in developing countries, including China [13]. Many molecular events are responsible for the initiation of gastric cancer and its progression. ATX, an extracellular lysoPLD, that catalyzes the conversion from LPC to LPA, exerts various biological effects through the lipid-signaling molecule, LPA [14,15]. OPN was shown to be frequently overexpressed in many human cancers, including lung cancer [16], colorectal cancer [17] and stomach cancer. OPN has been demonstrated to promote the survival of gastric cancer SGC7901 cells in the condition of cellular stresses induced by serum withdrawal or curcumin treatment [11]. Although several lines of evidence have suggested that the ATX-LPA axis and OPN can be used as tumor markers, the relationship between ATX and OPN is still not clear. In this study, we observed that LPA, ATX/LPC induced OPN expression (Figure 1), and OPN were required for the effect of the ATX-LPA axis on the human gastric cancer SGC7901 cells.

ATX, LPA, and OPN elicit a wide range of cellular responses, including cell proliferation and migration [11,5]. Taxol, known as paclitaxel, is a very important drug to promote apoptosis [12]. In our present study, Figure 4D showed that LPA and ATX/LPC was required in the protection against Taxol-induced apoptosis in SGC7901 cells. LPA and ATX/LPC also significantly promoted the migration of SGC7901 cells, but had no significant effects on the migration of OPN-deficient SGC7901 cells (Figure 4B, C), suggesting that OPN was indispensable for ATX/LPC-induced migration and OPN was required in the protective effect against Taxol-induced apoptosis in SGC7901 cells. It is likely that ATX generates LPA from LPC and then LPA displays protective effect on these cells, which requires further studies.

LPA mediates its activity through a series of G protein-coupled receptors [18]. Many gastric cancer cell lines have been shown to express at least one LPA receptor [19]. In our study, expression of the LPA2 receptor was up-regulated by ATX/LPC2 (Figure 2A-D), consistent with previous results that ovarian cancers showed markedly increased expression of LPA2 receptors [20]. Although Ki16425 (inhibitor of LPA receptors) is somewhat LPA-receptor specific with the order of inhibition of LPA1 > LPA3 >> LPA2, data from our experiment revealed that LPA2 possibly played more important roles in the process of ATX/LPC2- induced expression of OPN.

The extracellular signal-regulated kinase/mitogen-activated protein kinase (ERK-MAPK) and PI3K/Akt signaling pathways are evolutionarily conserved kinase modules that link extracellular signals to the machinery controlled fundamental cellular processes such as growth, proliferation, differentiation, migration and apoptosis; all are critical in human malignancies. In our study, phosphorylation of ERK and Akt were upregulated by ATX/LPC and LPA, and down-regulated by their inhibitors, PD98059 and LY294002. Our data clearly indicated that LPA-induced overexpression of OPN was mediated by either PI3K/Akt or ERK signaling pathways. ERK activates different transcription factors related to cell proliferation and survival such as Elk-1. As a major substrate of the MAPK family, Elk-1 plays a key role in cell differentiation, proliferation, tumorigenesis and apoptosis. Phosphorylation of Elk-1 appears to be critical for the activation of c-fos transcription [21]. Our results proposed that Elk-1 connected the intracellular signaling cascade of ATX-LPA2-OPN (Figure 3).

## Conclusions

Our overall results provided novel evidence that the ATX-LPA axis induces OPN expression via Akt and MAPK/ERK-mediated mechanisms, and OPN is required in migration induced by ATX-LPA axis, which protects against Taxol-induced apoptosis in SGC7901 cells.

## Methods

### Reagents

ATX (recombinant human ENPP-2/Autotaxin) and OPN antibody were purchased from R&D Systems (Minneapolis, MN, USA). Oleoyl-L-a-lysophosphatidic acid sodium salt (LPA), oleoyl-L-a-LPC, blasticidin, MAPK inhibitor (PD98059), PI3K inhibitor (LY294002), LPA receptor antagonist (Ki16425) and α-tubulin antibody (#T5168) were purchased from Sigma-Aldrich (Saint Louis, MO, USA). Monoclonal anti-p44/42 MAP kinase, anti-Phospho-p44/42 MAPK (Thr204/Tyr202), total-Akt and phospho-Akt (Ser473) antibodies were purchased from Cell Signaling Technology (Danvers, MA, USA). pFR-Luc plasmid and pFA2-Elk1 plasmid were purchased from Invitrogen (Carlsbad, CA, USA).

### Cell culture

Human gastric cancer SGC7901 cells were cultured in DMEM supplemented with 10% fetal bovine serum (FBS), penicillin (100 U/ml), streptomycin sulfate (100 μg/ml), and maintained at 37°C with 5% CO_2 _in a humidified incubator.

### Construction of OPN-siRNA expression plasmids

SiRNA against OPN was produced from Invitrogen (Carlsbad, CA, USA). The sequences of the selected region to be targeted by siRNA for OPN were:

SR54-1F:TGCTGAATTGACCTCAGAAGATGCACGTTTTGGCCACTGACTGACGTGCATCTTGAGGTCAATT;

SR54-1R: CCTGAATTGACCTCAAGATGCACGTCAGTCAGTGGCCAAAACGTGCAT

CTTCTGAGGTCAATTC;

SR54-2F: TGCTGTTAACTGGTATGGCACAGGTGGTTTTGGCCACTGACTGACCACC

TGTGATACCAGTTAA;

SR54-2R: CCTGTTAACTGGTATCACAGGTGGTCAGTCAGTGGCCAAAACCACCTGT

GCCATACCAGTTAAC;

SR54-3F: TGCTGAATCACATCGGAATGCTCATTGTTTTGGCCACTGACTGACAATG

AGCACCGATGTGATT;

SR54-3R: CCTGAATCACATCGGTGCTCATTGTCAGTCAGTGGCCAAAACAATGAG

CATTCCGATGTGATTC.

We used lipofectamine 2000 (Invitrogen, Carlsbad, CA, USA) to separately transfect three types of OPN constructs into SGC7901 cells. To select resistant colonies, 48 hours after transfection, cells were cultured in selective medium containing 3 μg/ml blasticidin (Sigma-Aldrich, Saint Louis, MO, USA). Blasticidin-resistant cells were maintained in culture medium supplemented with 3 μg/ml blasticidin for further analysis.

### Western blotting analysis

SGC7901 cells (1 × 10^6^) were treated with ATX(50 ng/ml), LPC(10 μM), LPA(10 μM), ATX(50 ng/ml)+LPC(10 μM)(ATX/LPC1) or ATX(50 ng/ml)+LPC(20 μM) (ATX/LPC2) for 24 hours, and then cells were lysed in RIPA buffer. Equal amounts of protein (60 μg) were electrophoresed on 12% SDS-PAGE gels and electrophoretically transferred to Immobilon-P membranes (Millipore, Bedford, MA, USA). Membranes were incubated overnight at 4°C with anti-OPN (1:100) or monoclonal anti-α-tubulin (1:5000) in TBST containing 1% BSA (w/v). The membranes were incubated for 2 hours with anti-rabbit or anti-mouse secondary antibodies, and the immune complex was detected using an ECL plus detection kit (Pierce, Rockford, IL, USA). The optical densities of each band and the density ratio of OPN to α-tubulin bands were calculated using a densitometer (Furi, Shanghai, China).

### Migration assay

Cell migration was performed using 24-well transwell migration plates (Corning Costar, Schiphol-Rijk, Netherland). The upper chamber was filled with 100 μl of cell suspension (1 × 10^5 ^cells) in DMEM/0.1% BSA. The lower chamber contained 600 μl of the DMEM/0.1% BSA with ATX (50 ng/ml), LPC (10 μM), LPA (10 μM), ATX/LPC2. The filters were fixed with methanol and stained with hematoxylin and eosin after 48h's incubation. Cells remaining on the top side of the filter were removed by soft mechanical scraping, and the number of cells migrating to the bottom of the filter was counted using a light microscope (in each chamber, six fields were counted at 200× magnification for each condition).

### RNA extraction and Real-Time PCR

Total RNA was extracted from SGC7901 cells incubated in 6-cm plates after 18 hours of stimulation with either ATX (50 ng/ml), LPA (10 μM), LPC (10 μM), ATX/LPC1, ATX/LPC2, or DMEM/0.1% BSA alone as a negative control, using Trizol (Invitrogen, Carlsbad, CA, USA) according to the manufacturer's instruction. Reverse transcription was performed using a RevertAidTM First Stand cDNA Synthesis Kit (MBI-Fermentas). The transcript levels for OPN, LPA1, LPA2, LPA3, LPA4 and β-actin were quantified by real-time PCR. The primers used were as follows:

β-actin-F: 5-TCACCAACTGGGACGACAT-3

β-actin-R: 5-GCACAGCCTGGATAGCAAC-3

OPN-F: 5-AGGCTGATTCTGGAAGTTCTG-3

OPN-R: 5-GCTTTCGTTGGACTTACTTGG-3

LPA1-F: 5-GGTGATGGGACTTGGAAT-3,

LPA1-R: 5-AAACCGTAATGTGCCTCT-3;

LPA2-F: 5-TGGCTCAACCCAACCAAC-3,

LPA2-R: 5-CCTCATTACCCAGTCATACCG-3;

LPA3-F: 5-TGCTTCCCTCACCAACTT-3,

LPA3-R: 5-CCGCAGGTACACCACAAC-3;

LPA4-F: 5-AGTTGTTGGGTTTATCATTC-3,

LPA4-R: 5-AAACAGGGACTCCAT TCT-3 [22];

### Flow cytometric analysis for apoptosis

SGC7901 cells were stimulated with the following treatments: Taxol (50 nM), or Taxol (50 nM) containing ATX (50 ng/ml), LPC (10 μM), LPA (10 μM), ATX/LPC2 for 24 hours. Cultured cells were collected with trypsin/EDTA and washed with PBS and stained with Propidium Iodide (PI) or FITC-conjugated antibodies. Fluorescence was quantified on 10,000 cells with FacsCalibur with Cellquest software (BD Biosciences, PharMingen)

### Luciferase (LUC) reporter assay

SGC7901 cells (1 × 10^5^) were transfected with pFR-Luc plasmid (reporter plasmid), or pFA2-Elk1 plasmid (fusion trans-activator plasmid). Six hours later, the cells were treated with the following drugs: ATX/LPC2, or ATX/LPC2 containing DMSO, Ki16425 (15 μM), PD98059 (50 μM), or LY294002 (50 μM) and incubated for an additional 24 hours. The cells were then harvested and tested using a Luciferase assay system (Promega, Madison, WI). An error bar was established to show the SD derived from three independent experiments.

### Statistical analysis

Data were analyzed using a two-tailed Student's t-test for single comparisons and by one-way analysis of variance for multiple group comparisons. Differences were considered significant at * P < 0.05 versus control.

## List of abbreviations

ATX: autotaxin; LPA: lysophosphatidic acid; LPC: lysophosphatidylcholine; OPN: osteopontin; MAPK: mitogen-activated protein kinase; ERKs: extracellular signal-regulated kinase; Elk-1: active MAPKs phosphorylate specific transcription factors; PI3K: phosphatidylinositol 3-kinase

## Competing interests

The authors declare that they have no competing interests.

## Authors' contributions

RZ carried out the cell studies, drafted the manuscript, and participated in western blot and real time-PCR. JW carried out the Migration assay. SM participated in the Flow cytometric analysis for apoptosis. ZH and GZ participated in the design of the study and performed the statistical analysis. All authors read and approved the final manuscript.
